# Synthetic Cationic Autoantigen Mimics Glatiramer Acetate Persistence at the Site of Injection and Is Efficacious Against Experimental Autoimmune Encephalomyelitis

**DOI:** 10.3389/fimmu.2020.603029

**Published:** 2021-01-18

**Authors:** Jimmy Y. Song, J. Daniel Griffin, Nicholas R. Larson, Matthew A. Christopher, C. Russell Middaugh, Cory J. Berkland

**Affiliations:** ^1^ Department of Pharmaceutical Chemistry, University of Kansas, Lawrence, KS, United States; ^2^ Department of Bioengineering, University of Kansas, Lawrence, KS, United States; ^3^ Department of Chemical and Petroleum Engineering, University of Kansas, Lawrence, KS, United States

**Keywords:** EAE, poly-lysine, multiple sclerosis, Copaxone^®^, glatiramer acetate, SC injection simulation

## Abstract

A synthetic peptide, K-PLP, consisting of 11-unit poly-lysine (K11) linked *via* polyethylene glycol (PEG) to proteolipid protein epitope (PLP) was synthesized, characterized, and evaluated for efficacy in ameliorating experimental autoimmune encephalomyelitis (EAE) induced by PLP. K-PLP was designed to mimic the cationic nature of the relapsing-remitting multiple sclerosis treatment, glatiramer acetate (GA). With a pI of ~10, GA is able to form visible aggregates at the site of injection *via* electrostatic interactions with the anionic extracellular matrix. Aggregation further facilitates the retention of GA at the site of injection and draining lymph nodes, which may contribute to its mechanism of action. K-PLP with a pI of ~11, was found to form visible aggregates in the presence of glycosaminoglycans and persist at the injection site and draining lymph nodes *in vivo*, similar to GA. Additionally, EAE mice treated with K-PLP showed significant inhibition of clinical symptoms compared to free poly-lysine and to PLP, which are the components of K-PLP. The ability of the poly-lysine motif to retain PLP at the injection site, which increased the local exposure of PLP to immune cells may be an important factor affecting drug efficacy.

## Introduction

Glatiramer acetate (GA), the active ingredient in Copaxone^®^, is currently one of the most popular treatments for relapsing-remitting multiple sclerosis (RRMS) due to its safety, ease of patient self-administration, and its effectiveness in reducing the relapse rate of RRMS patients (1, 2). Despite clinical and commercial success of GA, its full mechanism of action is yet to be completely understood. In the last few decades, many studies have tried to elucidate the immunomodulatory mechanism of GA, however, it has proven to be complicated and no unifying explanation exists (3–14). Systemic exposure of GA is near zero, suggesting a key part of the drug mechanism may be local to the site of administration. It is unclear if certain structural elements of GA may be extrapolated as a design principle for other autoimmune interventions such as antigen-specific immunotherapy.

GA consists of a broad population of peptides (average MW 5–9 kDa) comprising four amino acids (AKEY). Lysine is the predominant amino acid at ~34% of the molar mass, which imbues a strong cationic character (pI ~10). As a result, GA forms large, visible aggregates in the presence of glycosaminoglycans such as hyaluronic acid (HA) almost immediately upon contact (15). Approximately 30% of GA remained aggregated after 3 days according to *in vitro* subcutaneous (SC) injection simulation experiments. The large aggregated particles were also observed *in vivo* when GA was injected into the footpads of mice. Consequently, GA was able to persist at the injection site for a prolonged period of time. Considering the aggregation of GA likely occurs prior to all other immunological events, the ability to form aggregates *via* electrostatic interaction with glycosaminoglycans may be an important property that contributes to the mechanism of GA.

Cationic peptides such as poly-lysine were able to form visible aggregates in the presence of HA through electrostatic interactions similar to GA (15). In addition, poly-lysine-based polypeptides such as GEMSP have demonstrated to be a potential effective treatment for multiple sclerosis (16). GEMSP consists of a mixture of fatty acid linked poly-lysine (PLL), antioxidants-PLL, free radical scavengers-PLL, and amino acids-PLL. Even though the mechanism of action is not well understood, GEMSP almost completely ameliorated the symptoms of the animal model of multiple sclerosis called experimental autoimmune encephalomyelitis (EAE) and demonstrated the ability to preserve myelin integrity. Furthermore, GEMSP had shown no toxicity in both animals and humans. Based on these results, poly-lysine was chosen to be an integral part of our GA-mimic construct.

**Figure 1:**
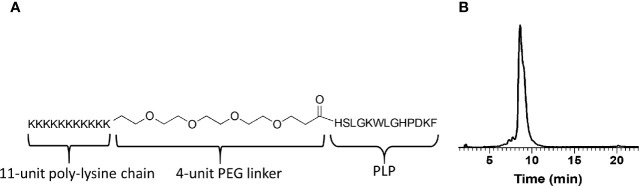
**(A)** The design and molecular structure of K-PLP. **(B)** The HPLC chromatogram of the purified product. The main product peak elutes at around 9 min on a Waters XBridge C_4_ column, 3.5 μm, 4.6 × 150 mm, linear gradient from 20% to 50% acetonitrile (+0.2% TFA) in water (+0.2% TFA) over 60 min, detection at 280 nm.

We set out to determine if the tissue-retention properties of GA could be combined with an antigen associated with RRMS. The relapse remitting form of EAE used to model RRMS in mice was induced using a specific epitope derived from myelin sheath, proteolipid protein 139–151 (PLP). To emulate GA properties, PLP was combined with an 11-unit poly-lysine “tail” (K11) to imbue an overall charge similar to GA (**Figure 1**). This custom-designed peptide (K-PLP) was anticipated to exhibit the two key features. First, aggregation with glycosaminoglycans was expected to be driven by the K11 portion, resulting in prolonged injection site retention and potential enrichment in draining lymph nodes. Second, antigen-specific immune responses driven by PLP localization were hypothesized to suppress the severity of EAE symptoms.

## Material and Methods

20 mg/ml solutions of Copaxone^®^ (glatiramer acetate) 1 ml pre-filled syringes from Teva Neuroscience, Inc. (Kansas City, MO) were donated by the University of Kansas Medical Center. For peptide synthesis, Fmoc-L-phenylalanine 4-alkoxybenzyl alcohol resin (0.3–0.8 meq/g, 100–200 mesh) and all amino acids were purchased from Chem-Impex International Inc. (Wood Dale, IL). Fmoc-NH-(PEG)_4_-CH_2_COOH (5,8,11,14-Tetraoxa-2-azahexadecanedioic acid) was purchased from PurePEG, LLC (San Diego, CA). Myelin proteolipid protein (PLP) epitope (HSLGKWLGHPDKF) and 9-unit poly-lysine were synthesized and purchased from Biomatik USA, LLC (Wilmington, DE). Scissor Cartridge packs containing HA based extracellular matrix (ECM) were obtained from Pion Inc. (Billerica, MA). Sulfo-Cyanine7 NHS ester was purchased from Lumiprobe (Hunt Valley, MD). PEG amine (5kDa) was obtained from Creative PEGworks (Chapel Hill, NC). All other analytical grade chemicals and reagents were purchased from MilliporeSigma (St. Louis, MO) and Fisher Scientific (Pittsburgh, PA) and were used as received. All mice used for the study were maintained in sterile housing under the veterinary supervision of the University of Kansas Animal Care Unit. All procedures were approved by the Institutional Animal Care and Use Committee.

### Peptide Synthesis

K-PLP (NH_2_-KKKKKKKKKKK-PEG-PEG-PEG-PEG-HSLGKWLGHPDKF-OH) was synthesized using CEM Liberty Blue™ Automated Microwave Peptide Synthesizer at 0.10 mmol scale using standard FMOC chemistry. Single coupling was performed on all amino acids except for the 11-unit poly-lysine tail, where double coupling was used to improve yield. The peptides were cleaved using a solution of 92.5:2.5:2.5:2.5 TFA : TIPS:H_2_O:DODT and the crude peptides where purified using preparative HPLC (Waters XBridge C4, 5 μm, 10 × 250 mm, linear gradient from 20 to 50% MeCN (+0.05% TFA) in H2O (+0.05% TFA) over 30 min, detection at 280 nm). The purified product was characterized using a Waters Alliance HPLC system (Waters XBridge C_4,_ 3.5 μm, 4.6 × 150 mm, linear gradient from 20% to 50% acetonitrile (+0.2% TFA) in water (+0.2% TFA) over 60 min, detection at 280 nm) and QTOF-Premier hybrid mass spectrometer (Micromass Ltd, Manchester, UK) operated in MS mode and acquiring data with the time of flight analyzer. The electrospray ionization (ESI) spectra were acquired at 15,625 Hz pusher frequency covering the mass range 300 to 5000 u and accumulating data for 3 s per cycle. Time to mass calibration was made with CsI cluster ions acquired under the same conditions. The resulting suite of charge states in the ESI spectrum were subject to charge state deconvolution to present a “+1” charge mass spectrum using the MaxEnt3 routine in MassLynx software. This routine removes the isotope cluster.

### Far-UV Circular Dichroism Spectroscopy

Circular dichroism was performed using a Applied Photophysical Chirascan (Applied Photophysics Ltd., Leatherhead,UK). One-millimeter pathlength quartz cuvettes were filled with 250 μl of K-PLP at 0.1 mg/ml in 150 mM citrate-phosphate buffer at pH 6. Cuvettes were placed in a six-position Peltier temperature controller (Quantum Northwest, Liberty Lake, WA). Samples and buffer were both measured at room temperature in triplicate from 195 – 260 nm and buffer subtraction was performed.

### Raman Spectroscopy and Dynamic Light Scattering

Raman and dynamic light scattering were performed with a Malvern Helix (Malvern Instruments, Malvern, UK). 20 μl of K-PLP at a concentration of 10 mg/ml in 150 mM citrate-phosphate buffer at pH 6 was loaded into a custom steel cuvette with quartz windows. Raman scattering from a 785 nm laser was collected in a backscattering geometry. Samples were measured in triplicate, each replicate consisted of 10 acquisitions of 10 second exposure each. The Zetasizer Helix Analyze software (Malvern Instruments) was used to buffer subtract, normalize to the phenylalanine peak (1003 cm-1), then baseline spectra for analysis. Dynamic light scattering autocorrelation functions were measured with a 632 nm laser with light collected in a 173° angle backscattering geometry. Autocorrelation functions were fit using the method of cumulants.

### Fluorescence Spectroscopy

Intrinsic tryptophan fluorescence spectra were obtained using a fluorescence plate reader as described by Wei et al (17). K-PLP was at a concentration of 10 mg/ml (same as Raman and DLS). Triplicate samples in a 384-well plate had silicone oil added atop to avoid sample evaporation during thermal ramps. The plate was centrifuged at 2,200 × g for 1 min to remove air bubbles. Samples were excited with 295 nm laser. Fluorescence emission between 300 and 400 nm was collected for 100 ms. Temperature was ramped from 10 to 90°C with an increment of 2.5°C per step and an equilibration time of 2 min at each step. The first moment (mean) of the fluorescence spectrum, λ_μ_, was calculated between 300 and 400 nm.

### Subcutaneous Injection Simulation and Release

The subcutaneous injection simulation was performed using the method previously described (15). 1 ml of 20 mg/ml K-PLP in 40 mg/ml mannitol solution was injected into Scissor cartridge containing 10 mg/ml 1.5–1.8 MDa hyaluronic acid (HA). Time points were collected over 3 days and concentration of K-PLP released into the chamber buffer was determined using a bicinchoninic acid assay (Micro BCA protein assay kit, Thermo Scientific, Waltham, MA) by constructing a calibration curve with K-PLP concentrations of 2, 5, 10, 20, 30, and 40 µg/ml. The concentration values were adjusted to account for K-PLP that was taken out during sampling and then converted to percentage of K-PLP released before plotted as a function of time.

### Fluorescent Labeling

GA, K-PLP, 5 kDa PEG amine, and PLP were fluorescently labeled using sulfo-cyanine 7 NHS Ester (sodium 1-(6-((2,5-dioxopyrrolidin-1-yl)oxy)-6-oxohexyl)-3,3-dimethyl-2-((E)-2-((E)-3-(2-((E)-1,3,3-trimethyl-5-sulfonatoindolin-2-ylidene)ethylidene)cyclohex-1-en-1-yl)vinyl)-3H-indol-1-ium-5-sulfonate). For GA, 5kDa PEG amine, and K-PLP, 5 equivalents (7000 Da used as MW of GA) of compound were reacted with 1 equivalent sulfo-cyanine 7 NHS Ester in 50 mM HEPES buffer pH 7.5 with 20% DMSO. The reaction was performed at room temperature for 4 h protected from light with stirring. To separate labeled drug from excess dye, the reaction mixture was placed into dialysis cassettes with 2 kDa MWCO and dialyzed in water with buffer change every 12 h for 72 h. To label PLP, equal molars of PLP and sulfo-cyanine 7 NHS Ester were reacted in dry DMSO under nitrogen. N,N-Diisopropylethylamine (41 equivalents) was added to PLP and allowed to stir before adding the sulfo-cyanine 7 NHS Ester dropwise. The reaction was performed at room temperature for 4 h protected from light with stirring. Purification was performed using preparative HPLC (Waters XBridge C4, 5 μm, 10 × 250 mm, linear gradient from 15 to 50% MeCN (+0.05% TFA) in H2O (+0.05% TFA) over 30 min, detection at 280 nm). The final purified products were all lyophilized. The number of dye labeled onto each compound was determined by constructing a calibration curve based on the fluorescence of Sulfo-Cyanine7 NHS ester (Ex:760 nm, Em: 782 nm) at various concentrations and comparing the fluorescence of the labeled product to the calibration curve. No significant deviations of the excitation and emission spectra between each of the compounds and free dye were found. Herein, the labeled compounds will be referred to as cy7-GA, cy7-K-PLP, cy7-PEG and cy7-PLP. The amount of dye labeled onto each compound were reported as μmol of dye per g of compound, and they were determined to be 48.1 ± 1.7 μmol/g for Cy7-K-PLP; 733.4 ± 29.4 μmol/g for cy7-PLP; 39.8 ± 0.3 μmol/g for cy7-GA; 68.9 ± 11.9 μmol/g for cy7-PEG.

### In Vivo Efficacy Study

*In vivo* efficacy of K-PLP was assessed using the EAE animal model induced in female 4-6-week old SJL/J (H-2) mice (20–25 g, Envigo, Indianapolis, IN). All protocols were approved through the University’s Institutional Animal Care and Use Committee, and all animals were housed in pathogen-free conditions. EAE induction and treatment schedule were based on methods previously described (18–20). An emulsion was prepared containing 200 μg free PLP in PBS emulsified with Complete Freund’s Adjuvant (CFA) containing 4 mg/ml heat killed M. Tuberculosis strain H37RA. On day 0, this emulsion was administered to mice *via* 50 μl SC injections above each shoulder and hind flank resulting in a total emulsion injection volume of 200 μl per mouse. Additionally, on day 0 each mouse received a 100 μl intraperitoneal injection of pertussis toxin at 100 ng/ml. This administration of pertussis toxin was repeated on day 2. Beginning on day 7, disease severity was monitored daily through the use of a symptom scoring system as follows: 0, no clinical disease symptoms; 1, weakness or limpness of the tail; 2, weakness or partial paralysis of one or two hind limbs (paraparesis); 3, full paralysis of both hind limbs (paraplegia); 4, paraplegia plus weakness or paralysis of forelimbs; 5, moribund (euthanasia necessary). Mouse weight was also recorded daily throughout the study. *In vivo* treatment groups consisted of 6 mice per group. Treatments were administered in 100 μl SC injections formulated in 40 mg/ml mannitol. Injections were administered between the shoulder blades. Copaxone and K-PLP were administered at 4.5 mg/ml while 9-unit poly-lysine was administered at the molar equivalent to K-PLP injections, resulting in a concentration of 1.7 mg/ml. Treatment injections were performed on days 4, 7, and 10 following EAE induction. Mouse weights were recorded daily beginning on day 0 and clinical scores were recorded daily beginning on day 7. Statistical analysis was performed using two-way analysis of variance (ANOVA) on the clinical scores and weights, one-way ANOVA on the cumulative clinical scores, followed by Turkey comparison tests for all. Statistical significance for all analyses was set at p<0.05. All statistical analyses were performed using GraphPad Software (GraphPad Software Inc.).

### In Vivo Migration Imaging

Similar imaging method as previously described was used (15). Three female SJL/J mice (20-25 g, Envigo, Indianapolis, IN) were used for imaging, one each for K-PLP, PLP, and GA. Unlabeled K-PLP and unlabeled GA were mixed with cy7-K-PLP and cy7-GA, respectively, to a final concentration of 20 mg/ml with the amount of dye normalized to the equivalent of 25 μM of free cyanine 7 dye. Similarly, unlabeled PLP was mixed with cy7-PLP to a concentration that is molar equivalent to 20 mg/ml of K-PLP with the amount of dye normalized to the equivalent of 25 μM of free cyanine 7 dye. Each mouse was injected with 10 μl of their respective treatment at the center of the footpad every hour for 4 h in the following order: left forelimb at the 1-h time point, right forelimb at the 2-h time point, left hindlimb at the 3-h time point, and right hindlimb at the 4-h time point for K-PLP; right forelimb at the 1-h time point, left forelimb at the 2-h time point, right hindlimb at the 3-h time point, and left hindlimb at the 4-h time point for both GA and PLP. Injecting at opposing limbs allows direct comparison between K-PLP with GA/PLP at each time point. fluorescent images were taken using a MaestroFlex whole body imager (Cambridge Research and Instrumentation, Woburn, MA) employing an excitation filter of 710–760 nm and a longpass emission filter of 800 nm. The draining lymph nodes near each site of injection was later resected and imaged to determine whether the GA or PEG is draining toward this lymph node.

### Cell Internalization Assay

Splenocytes from mice induced with experimental autoimmune encephalomyelitis (EAE) were harvested on day 14 post-induction from the spleen. Nine replicates (per group) of approximately one million cells were plated into each of the 96 wells in 100 μl cRPMI medium (RPMI medium with 10% FBS and 1% penicillin streptomycin). Cy7-K-PLP, cy7-PLP, and cy7-PEG were prepared in cRPMI to a concentration of 50 μM. To each replicate of cells, 100 μl of the 50 μM compound solution was added to a final concentration of 25 μM per well. The cells were then incubated for 1 h at 37°C with 5% CO_2_. After incubation, the 96 well plate was spun at 300 g for 10 min and the supernatant was aspirated. To the resulting pellet, a fresh 200 μl of cRPMI medium was added and mixed to resuspend the cells. To quantify fluorescence, each compound was serial diluted with cRPMI from 25 μM to 0.01 μM (2 fold dilution each step) and a fluorescence-concentration calibration curve was constructed. The fluorescence experiments were performed using Synergy™ H4 Microplate Reader (BioTek, Winooski, VT) with excitation wavelength of 760 nm, emission of 782 nm (the excitation and emission max of sulfo-cyanine 7). The resulting fluorescence intensity of the cells treated with compounds were compared to their respective calibration curve.

## Results

### Peptide Design, Synthesis, and Characterization

K-PLP consists of an 11-unit poly-lysine and MS antigen PLP _139-151_ bridged by a flexible region comprised of four units of PEG (**Figure 1A**). The design of this peptide intends to mimic the cationic properties of GA and additionally introduces antigen specificity. Meanwhile, this clearly defined sequence allows K-PLP to avoid many of the complexities associated with the sequential randomness and broad MW distribution of GA. By including an 11-unit poly-lysine region on K-PLP, the isoelectric point of K-PLP is modified to approximately 11.4 and the net charge at pH 7 is approximately 12.2, which is similar to GA (isoelectric point of 9.8, net charge at pH 7 of 12.0).

The peptide was synthesized on a microwave peptide synthesizer using standard FMOC chemistry and purified through RP-HPLC. The purified product was characterized using analytical HPLC and QTOF mass spectrometer. The HPLC chromatogram (**Figure 1B**) showed that the main peak (~9 min) has a shoulder, suggesting the co-elusion of unresolved compounds. This slight impurity was confirmed using mass spectrometry where in addition to the expected monoisotopic mass of 3,177.9, another peak at 3,306.0 was present and its relative abundance is ~20% (relative abundance of 3,177.9 peak at 100%). The difference in the mass of 128 indicated the presence of an additional lysine. Taken together, the purified K-PLP contained the expected product as well as another product that has an additional lysine. Although the additional lysine introduces another overall positive charge, being located in the poly-lysine region would simply enhance the electrostatic interaction between the peptide and the ECM and should not significantly affect antigen (PLP) recognition.

### Structural Characterization of K-PLP

**Figure 2:**
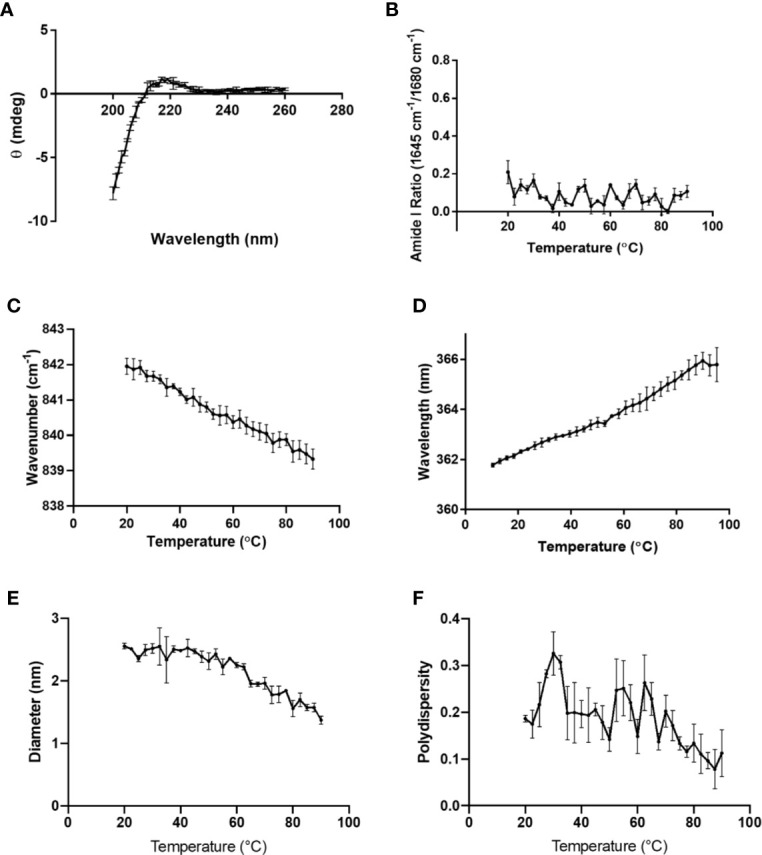
**(A)** CD spectrum of 0.1 mg/ml K-PLP performed at room temperature and pH 6. Raman spectroscopy was performed on 10 mg/ml K-PLP at pH 6 over a temperature range of 20°C –90°C, **(B)** shows the amide I ratio of 1,645 over 1,680 cm^-1^, which represents the ratio of α-helix to β-sheet, and **(C)** is the Raman tryptophan (840 cm^-1^) moment changes over temperature. **(D)** shows the moment change of tryptophan fluorescence λ_max_ over a temperature range of 20°C –90°C. **(E)** The plot of particle size versus change in temperature was obtained using DLS and its polydispersity is shown in **(F)**.

To determine whether K-PLP possess higher-ordered structures in solution, multiple characterization techniques were employed (15). The CD spectrum of K-PLP from 200 nm to 260 nm was shown in **Figure 2A**. Although the spectrum was obtained from 195 nm, significant noise was present <200nm. The CD spectrum showed a slight positive band near 218 nm, and even though no distinctive negative band was present at 198 nm, the spectrum resembled a random coil (21). Raman spectroscopy also supported the lack of distinct secondary structure. The ratio of Raman peaks of 1,645 cm^-1^ over 1,680 cm^-1^ (**Figure 2B**), which represents α-helical and β-sheet structure respectively, remained relatively constant over the temperature ranges of 10°C to 90°C. This implied that the secondary structure of K-PLP was not sensitive to changes in temperature over a broad range. Tertiary structure was examined through both Raman (Trp, 840 cm^-1^) and intrinsic fluorescence (Trp λ_max_). The moment (mean spectral center of mass)-temperature plot (**Figures 2C, D**) from both techniques demonstrated that a thermally induced unfolding event was absent, which can be explained by the absence of tertiary structure. Furthermore, the λ_max_ at 25 °C of Trp intrinsic fluorescence was 355 nm, which was indicative that the Trp was fully solvent accessible (22). These results holistically supported that K-PLP may be a random coil that also lacked tertiary structure in solution.

DLS particle sizing results suggested that the mean size of K-PLP was around 2 nm, and in general the polydispersity values were less than 0.3 (**Figures 2E, F**). The average diameter appeared to decrease as temperature increased. However, since K-PLP was determined to lack higher-ordered structure, the decrease in size was unlikely to be the result of a thermally induced unfolding event. Rather, it was more likely caused by the increased rate of diffusion at higher temperatures.

### Subcutaneous Injection Simulation

**Figure 3:**
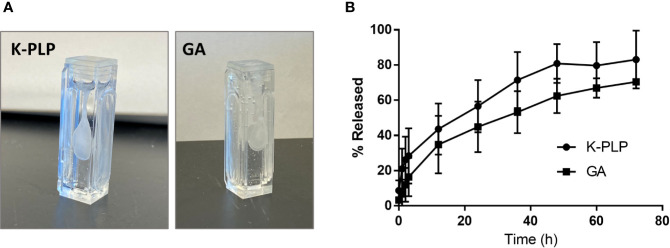
**(A)** Formation of aggregates was observed immediately upon injection of 1 mL of 20 mg/ml of K-PLP or 20 mg/ml of glatiramer acetate (GA) into 10 mg/ml hyaluronic acid (HA). **(B)** The percentage of K-PLP released into the *in vitro* model chamber over a period of 3 days compared to GA.

The release of K-PLP was simulated using the Scissor (Pion Inc.) subcutaneous injection site simulator system in a similar manner as previously described (15). As with GA, visible aggregates were formed immediately upon the injection of 20 mg/ml K-PLP into 10 mg/ml HA solution (1.5–1.8 MDa), which was used to mimic the ECM found in the SC space (**Figure 3A**). The release profile generated by monitoring drug release into the chamber showed that approximately 20% of K-PLP was released steadily into the chamber over the first 3 h and plateaued around 80% after 2 days (**Figure 3B**). This suggested that about 20% of K-PLP still remained in the cartridge at the end of the three-day experiment, and indeed, visible aggregates were found at the bottom of the cartridge. Compared to the release profile of GA, K-PLP released at a similar rate as GA (15).

### In Vivo Migration Imaging With Fluorescently Labeled K-PLP, Glatiramer Acetate, and Proteolipid Protein

**Figure 4:**
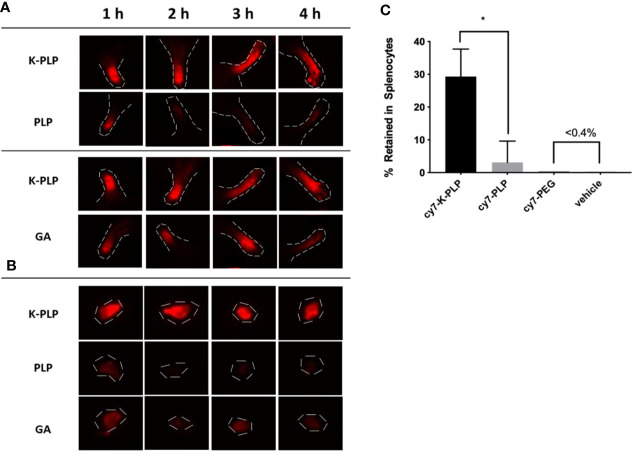
**(A)** Retention of fluorescently labeled cy7-K-PLP at the footpads compared to cy7-PLP alone or cy7-GA for 4 h. Noticeable longer retention is seen in K-PLP compared to PLP, and similar retention is seen compared to glatiramer acetate (GA). **(B)** Resected Auxiliary lymph nodes from each limb at different time points and different treatment. K-PLP appears to be more strongly retained in the lymph nodes compared to both GA and PLP. **(C)** Percentages of different treatments internalized by the splenocytes. Significantly more cy7-K-PLP was up taken than cy7-PLP and controls. *P < 0.05.

In our previous study, GA was able to remain at the site of injection for a longer period of time compared to a similarly sized PEG chain, which was used as a control (15). In this study, K-PLP was compared to GA as well as PLP (the antigen without poly-lysine “tail”). To ensure comparability, the unlabeled compounds were mixed with their labeled counterpart to obtain a final dye concentration of 25 μM for all solutions. To each mouse, 10 μl of the corresponding compound was injected into a different footpad every hour for 4 h. Examining the near-IR fluorescent images of the footpads, the fluorescence intensity of K-PLP did not significantly decrease over the course of 4 h (**Figure 4A**). Compared to K-PLP, the fluorescence intensity of PLP decreased significantly after just 1 h and became barely visible by the end of 4 h. Fluorescence intensity of GA, as expected, also did not significantly decrease over the course of the experiment and is comparable to K-PLP. These images suggested that the poly-lysine “tail” of K-PLP did aid in the retention of the drug at the site of injection and that retention is comparable to that of GA.

The draining lymph nodes near each of the limb were resected to determine if drainage into the lymph had occurred. The fluorescent images of the lymph nodes showed that drainage toward the lymph for all three compounds did occur, as the lymph nodes at 1 h all had fluorescence to some degree (**Figure 4B**). The fluorescence intensity of K-PLP lymph nodes however, remained relatively unchanged over 4 h, and was the brightest compared to PLP and GA. Contrarily, fluorescence of PLP lymph nodes became weakly visible after 1 h. Fluorescence of GA lymph nodes were noticeable at all time points but were significantly weaker than that of K-PLP. These results provided evidence that K-PLP may be able to retain in the lymph nodes even better than GA.

Transport to lymph nodes was further supported by the splenocyte internalization assay. Splenocytes were taken from mice induced with EAE and were incubated with 25 μM of cy7-K-PLP, cy7-PLP, and cy7-PEG for 1 h prior to centrifuging and aspirating the supernatant. The resulting pellet was resuspended and their fluoresce intensity was measured to determine how much of each labeled compound was retained by the cells. Cy7-PLP, the same antigen used to induce EAE, and cy7-PEG, a neutrally charged peptide that has approximately the same average molecular weight as K-PLP, both served as controls for the study. The splenocytes were able to retain (**Figure 4C**) 29.3 ± 8.4% of cy7-K-PLP fluorescence, 3.1 ± 6.5% of cy7-PLP fluorescence, and less than 0.4% of both cy7-PEG and the vehicle (cRPMI media only) fluorescence. The significant internalization of cy7-K-PLP by the splenocytes implied that cy7-K-PLP was actively transported into the lymph nodes, which was demonstrated in **Figure 4B**.

### In Vivo Efficacy

**Figure 5:**
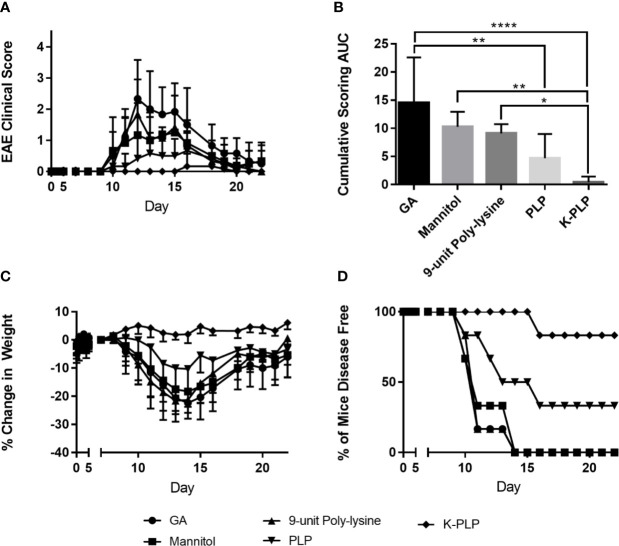
**(A)** Average clinical score of each treatment group from day 0 to day 25. Disease symptom onset appears to be the around day 9, reaching peak disease on day 14, and gradual remission thereafter. The scoring system is as follows: 0, no clinical disease symptoms; 1, weakness or limpness of the tail; 2, weakness or partial paralysis of one or two hind limbs (paraparesis); 3, full paralysis of both hind limbs (paraplegia); 4, paraplegia plus weakness or paralysis of forelimbs; 5, moribund (euthanasia necessary). **(B)** The average cumulative experimental autoimmune encephalomyelitis **(EAE)** clinical score from day 0 to day 25 for each treatment group. *p < 0.05, **p < 0.01, ***p < 0.001, and ****p < 0.0001. **(C)** % weight change of each treatment group normalized to the weight on day 7, when all mice are healthy. **(D)** The percentage of disease-free mice for all treatment groups compared.

To determine if K-PLP could be efficacious in ameliorating disease, K-PLP, GA, PLP_139-151_, and a 9-unit poly-lysine (similar to the poly-lysine chain found on K-PLP) were used to treat mice induced with EAE using PLP _139-151_. Remarkably, mice treated with K-PLP had the lowest clinical score (**Figures 5A, B**), resulted in the least weight decrease (**Figure 5C**), and had the highest percentage of disease-free mice (**Figure 5D**). Conversely, mice treated with 9-unit poly-lysine, GA, and PLP followed a similar disease progression as the control (mannitol), and any deviations from the control were not statistically significant. Based on the cumulative clinical scores (**Figure 5B**), K-PLP showed significant improvement over 9-unit poly-lysine, but not PLP. However, based on weight change data, K-PLP was more efficacious than PLP; from day 13 to day 17, which covered the span of peak disease, K-PLP treated mice experienced no significant weight loss compared to both 9-unit poly-lysine (p<0.0001) and PLP (p<0.01). Change in weight may be a better parameter than clinical scores in determining the effectiveness of K-PLP since this measure is not subjective like clinical scoring. The efficacy study suggested that K-PLP is more effective at ameliorating clinical symptoms of EAE than both of its individual parts (poly-lysine and PLP).

## Discussion

Compared to GA, which was shown to contain alpha helical and beta sheet structure in solution, K-PLP did not appear to have any form of ordered structure in solution (15). However, the lack of ordered structure did not seem to hinder the ability of K-PLP to form visible aggregates similar to GA (**Figure 3A**). This further reinforces the idea that the formation of aggregates is the result of electrostatic interaction between the positively charged lysine residues and the negatively charged glycosaminoglycan polymers. The native in-solution structure of GA and K-PLP alike does not appear to play a significant role in the phenomenon. K-PLP and GA have also demonstrated their ability to retain at the site of injection for longer period of time than PLP (**Figure 4**). Wu et al (23). performed a similar experiment where they injected fluorescently-labeled proteins of sizes ranging from 23 to 143 kDa into the footpads of mice. They discovered a significant positive correlation between size of protein and retention time at the site of injection. Therefore, it is unsurprising that compounds such as K-PLP and GA, which were able to form visible aggregates in the presence of glycosaminoglycans, were retained longer than compounds like PLP that did not form aggregates. The release profiles (**Figure 3B**) generated from SC release simulation using the Scissor instrument further substantiates the dominant role of aggregation caused by electrostatic interaction play in the retention of compound at the site of injection. Even though on average K-PLP is a smaller molecule than GA based on molecular weight and DLS particle sizing (**Figure 2E**), the release profile of the two were remarkably similar, suggesting the in-solution molecular weight was no longer an important factor that influenced drug diffusion once aggregates were formed at the injection site.

Even though K-PLP had shown that it behaved similarly to GA at the site of injection, the more important question to answer was whether K-PLP could be efficacious in ameliorating the disease. In the past work, we observed that highly concentrated depot retention of autoantigen is potent against EAE (24). In this work, we demonstrated that the clinical outcomes of K-PLP treated mice were significantly better than 9-unit poly-lysine or PLP alone. This suggested that injection site aggregation (9-unit poly-lysine, and GA) and having the antigen alone (PLP only) are decidedly inferior to pieces together. This observation further suggested that the induced aggregation and retention of disease-specific antigen is a viable therapeutic mode of action. Falk et al (25). reported that 16-mer oligomeric PLP constructs were able to significantly ameliorate EAE and they suggested that multimers of the disease-peptide were able to overstimulate the auto-reactive T-cells, which ultimately resulted in their apoptotic elimination. Similarly, Wegmann et al (26, 27). have shown that an 8-mer oligomeric-PLP attached to a central poly-lysine core (K_4_-K_2_-K) were able to alleviate symptoms of EAE. The purpose of the poly-lysine core is simply to increase solubility, nevertheless, it may have resulted in injection site retention through similar mechanisms as K-PLP. Although exact mechanisms are unknown, these works have evidenced that trafficking of peptide-specific encephalitogenic cells into the central nervous system is diminished by the oligomeric-PLP peptides. The commonality between these studies is that the larger multimer structures of PLP appear to be more therapeutic than peptide chains of PLP alone. Even though K-PLP is a linear chain with only one PLP epitope, the ability to form higher order aggregates allows the peptide to build potentially even larger multimers than the oligomers of PLP. These large aggregate structures embedded with disease-antigen may be able to exhaust the responses from both the innate and adaptive immunity, which ultimately may have resulted in disease amelioration (24, 28).

In this study, GA did not significantly improve the clinical outcomes of EAE mice compared to the control. This observation is contrary to many of the previous studies on GA (3–6, 29–33), though it is important to note differences in experimental conditions. Teitelbaum et al (33). initially observed that GA suppressed EAE in guinea pigs, however, other than using different species, they also induced EAE using MBP instead of PLP. GA was initially designed to mimic MBP and cross-reactivity between GA and MBP has been reported (6, 31). As a result, GA was logically more efficacious in such model. In a later paper, Teitelbaum et al (32). demonstrated that GA was also able to suppress EAE induced by PLP in mouse model, but only by co-injecting GA with PLP during induction. In a more recent study, Aharoni et al (30). have shown the neuroprotective effects of GA in EAE mouse models induced with PLP, however, their dosage was nearly 4 fold higher than our study and also with higher dosing frequency. Thus, the lack of efficacy of GA in our model may have resulted from multiple factors such as dosage, timing of treatment, and relevance to the encephalitogenic agent used to induce EAE.

Splenocytes were found to significantly uptake more K-PLP than PLP or PEG. This observation may help in understanding some of the underlying immunomodulatory mechanisms of K-PLP. Cationic peptides like poly-lysine have been known to facilitate the penetration through cell membranes, and they have shown to be useful in drug delivery applications such as gene delivery (34, 35). Likewise, the poly-lysine chain found on K-PLP may have facilitated the internalization of K-PLP by innate immune cells and subsequently transported to the lymph nodes, where antigen-specific immunomodulation could occur. Only when both poly-lysine chain (to facilitate internalization and transport) and the antigen epitope (to prime for the correct cell response signal) are present can a therapeutic effect be seen.

## Conclusion

In this study, we have successfully constructed, characterized, and tested a model peptide consisting of 11-unit poly-lysine connected to MS/EAE antigen PLP designed to mimic GA’s behavior at the site of injection. The peptide was characterized using biophysical techniques such as Raman spectroscopy, CD, intrinsic fluorescence, and DLS; the results showed that unlike GA, K-PLP lacked higher-ordered structure and is smaller in size (~2nm) in solution. Regardless, K-PLP was able to form visible aggregates in the presence of HA and its release profile generated through SC injection simulation showed a similar trend to GA. When K-PLP was injected into the footpads into mice, it was able to persist at the site of injection longer than the antigen PLP alone and at a similar rate to GA. In the efficacy study where EAE mice were treated with different compounds, significant improvements in clinical outcomes were observed in K-PLP-treated mice compared to GA, PLP, and poly-lysine-treated mice. Taken together, K-PLP demonstrated the importance of antigen localization at the site of injection and ultimately provided another piece of puzzle in the ever-evolving understanding of the immunomodulatory mechanisms of the glatiramoid class of drugs.

## Data Availability Statement

The original contributions presented in the study are included in the article/supplementary material; further inquiries can be directed to the corresponding author.

## Ethics Statement

The animal study was reviewed and approved by the Animal Care Unit of the University of Kansas.

## Author Contributions

JS designed, synthesized, and characterized the K-PLP peptide and all fluorescently labeled compounds. NL designed and performed the biophysical characterizations of K-PLP. JS designed and performed *in vitro* modeling and footpad retention studies. JG designed and performed the cell internalization assay. JS, JG, and MC designed and performed the *in vivo* efficacy studies. All data analyses were performed by the appropriate author(s). JS drafted the manuscript. All authors contributed to the article and approved the submitted version.

## Funding

This work was funded by the National Institutes of Health Biotechnology Training Grant (NIH0073415, 5T32GM008359-26), National Institutes of Health COBRE Chemical Biology of Infectious Diseases grant (P20-GM113117), PhRMA Foundation Postdoctoral Fellowship in Pharmaceutics, and the Madison and Lila Self Graduate Fellowship and Stella Fellowship from the University of Kansas.

## Conflict of Interest

The authors declare that the research was conducted in the absence of any commercial or financial relationships that could be construed as a potential conflict of interest.
